# A mixed methods exploratory study of women’s relationships with and uses of fertility tracking apps

**DOI:** 10.1177/2055207618785077

**Published:** 2018-07-25

**Authors:** Katie Gambier-Ross, David J McLernon, Heather M Morgan

**Affiliations:** 1 School of Health in Social Science, University of Edinburgh, Edinburgh, UK; 2 Institute of Applied Health Sciences, University of Aberdeen, Aberdeen, UK

**Keywords:** Digital health, fertility, menstrual tracking, mixed methods, period tracking apps

## Abstract

Digital self-tracking is rising, including tracking of menstrual cycles by women using fertility tracking apps (FTAs). However, little is known about users’ experiences of FTAs and their relationships with them. The aim of this study was to explore women’s uses of and relationships with FTAs. This exploratory study employed a mixed methods approach, involving the collection and analysis of an online survey and follow-up interviews. Qualitative analysis of survey and interview data informed hypothesis development. Online surveys yielded 241 responses and 11 follow-up interviews were conducted. Just over a third of women surveyed had experience of using FTAs (89/241) and follow-up interviews were conducted with a proportion of respondents (11/241). Four main motivations to use FTAs were identified: (a) to observe cycle (72%); (b) to conceive (34%); (c) to inform fertility treatment (12%); and (d) as contraception (4%). Analysis of the free-text survey questions and interviews using grounded theory methodology highlighted four themes underpinning women’s relationships with FTAs: (a) medical grounding; (b) health trackers versus non-trackers; (c) design; and (d) social and ethical aspects. Participants who used other health apps were more likely to use FTAs (*p* = 0.001). Respondents who used contraception were less likely to use FTAs compared with respondents who did not use contraception (*p* = 0.002). FTA usage also decreases (*p* = 0.001) as age increases. There was no association between FTA usage and menstrual status (*p* = 0.259). This research emphasises the differing motivations for FTA use. Future research should further explore the diverse relationships between different subgroups of women and FTAs.

## Introduction

### Overview

Fertility tracking apps (FTAs) are smartphone or tablet applications that allow users to record the dates of their menstrual cycles, as well as additional information, such as mood, symptoms, sexual activity and medications. FTAs claim to predict fertility windows based on menstrual cycle data, which may help women who are either wishing to conceive or trying to avoid conceiving. FTAs may also assist women and their fertility consultant when planning start dates for fertility treatment as they can provide menstrual cycle information to review at their fingertips, which may offer insights or patterns that memory or verbal testimony alone could not.

### Health apps

With increased smartphone usage, people have easier access to health information and, in turn, there has been an increase in people seeking health information electronically, developers providing health information digitally and health professionals recommending health apps to patients as part of preventive medicine and self-care regimens.^1^ Health apps include smartphone applications that can be downloaded and allow those who use them to track, monitor and act on their physiological, psychological or social health data. This is consistent with the emphasis of health professionals in recent years to assist people to self-manage their health, especially long-term conditions.

Health apps can empower users to monitor and manage their own health^2^ and using them is becoming common practice. For example, a study in the USA found that 58% of mobile phone users have downloaded a health app.^3^ But it is not clear to what extent the available technologies are based on clinical evidence or integrated into formal health and social care services. Whilst information collected via these technologies might be informally shared with healthcare providers or used as tools to comply with medical advice, they are not yet considered medical-grade devices and are not governed by any regulatory guidelines. However, they are beginning to be recommended by some health professionals.^4,5^

A fast-emerging niche of the ‘quantified self’ movement (also known as ‘lifelogging’, which involves individuals incorporating technology into data acquisition on aspects of their daily life) is fertility tracking.^6^ Apart from ‘activity trackers’, FTAs are the most frequently downloaded health app in the Apple App Store.^7^ Fertility tracking may also assist those under ‘expectant management’ (clinically advised to continue trying to conceive for a prescribed period) to time sexual intercourse around the fertile window.

### History of fertility tracking

Although FTAs are a relatively new concept, the act of fertility tracking itself is not. Women have historically used the fertility awareness method (FAM) to document their menstrual cycles with pen and paper but now, with increasing smartphone use, it is possible to track via apps. It is often used to plan activities around menstrual bleeding and sexual intercourse around fertile days to plan or avoid pregnancy. Comparative efficacy of the FAM of contraception remained unknown.^8^ However, the FAM is a less effective form of contraception than other methods such as the intrauterine device (IUD).^9^ The FAM method requires women to monitor changes such as basal body temperature, cervical mucus or cervical position.

### FTAs

There is emerging research on the efficacy of FTAs as methods of contraception.^10^ For example, a recent study found that the efficacy of a contraceptive mobile application is higher than usually reported for traditional fertility awareness-based methods and suggested that the application may contribute to reducing the unmet contraceptive planning needs for women.^11^ Another study based on the data of a web-based service for the FAM discussed the advantages of adapting natural family planning to information technology-based methods by allowing fertility to be viewed as a simple bar chart.^12^

However, the accuracy of web-based and app-based platforms in general^13^ and for predicting fertile windows to aid conception^14,15^ has been questioned. One study, which evaluated the features and functionality of FTAs highlighted the lack of health professional and evidence-based input.^16^ The importance of substantiating claims around fertility and contraception with clinical evidence is highlighted.^11^ However, some apps have been found to be more accurate than others, particularly those that use symptothermal methods to calculate fertility windows.^17^

### FTA market and regulation

Currently, there is no single regulatory body (local, national or international) approving FTAs before they enter the market; therefore, there is a huge variance in how ‘medically sound’ each app is. Women could have vastly different experiences using these apps depending on which one they use and how they use it. It is not known, perhaps beyond commercially sensitive market research conducted by provider companies, how women select an app and whether clinical endorsement is important to users.

Whilst conducting this research, it was announced that Natural Cycles (www.naturalcycles.com/en) had been approved as a recommended form of contraception in the European Union by the UK’s Medicines and Healthcare products Regulatory Agency.^18^ This is the first step towards FTAs being approved and recommended by healthcare professionals as a form of contraception.

### Potential implications of app usage

Although an interest in digital data in popular and research cultures is now evident, we still do not know enough about how people interact with and use the digital health data that they generate.^19^ It is important to improve our understanding of the uses of and relationships with health and digital technologies as we live in a world where it is almost impossible to avoid interactions with them. Research about FTAs may enable new insights into understudied aspects of how female bodies are experienced and understood. FTAs may improve women’s ability to manage their reproductive health information and communicate with their doctors and partners.

### Research aim

The aim of this exploratory study was to explore women’s uses of and relationships with FTAs in order to independently and scientifically inform the design and development of the next generation of FTAs.

## Methodology

### Study design

A mixed methods study, involving collection, analysis and synthesis of data obtained through an online survey using closed and open questions (quantitative and qualitative) and follow-up interviews (qualitative), was employed. Both quantitative and qualitative analysis informed hypothesis development. Hypotheses were developed following the analysis of survey data (descriptive statistics, grounded theory) and interview data (grounded theory), at which point the quantitative data were tested against the hypotheses for significance. Data collection involved a sequential approach (see Appendix A).

#### Online survey

Following ethical approval by the University of Aberdeen’s College Ethics Review Board (CERB), a survey was created using SurveyMonkey (www.surveymonkey.co.uk), a secure online platform, in March 2017 (see Appendix B for full study protocol). Participants were recruited online, primarily via social media platforms Facebook (www.facebook.com) and Twitter (www.twitter.com) The online survey was shared on the social media accounts of the Fertility Network UK charity and the research team (see Appendix C). It was also distributed via email to staff and students at the University’s School of Medicine, Medical Sciences and Nutrition and captured opt-in respondents’ demographic characteristics, smartphone usage, general fertility, FTA usage and experiences using FTA data (see Appendix D for survey questions). The survey was online for five days (116 hours). Survey responses were anonymous unless a participant volunteered to take part in a follow-up interview, in which case a name (did not have to be the respondent’s actual name) and email address was collected to facilitate follow-up contact. These methods of recruitment were chosen to allow for a more diverse sample.^20^ Online distribution of surveys is becoming popular and increasingly trusted.^21^ Social media was used because of its ability to collect data rapidly and cost-effectively. Moreover, these methods allow researchers to directly access prospective study participants who may be otherwise difficult to reach (because of their low prevalence or remote location).^22^ Informed consent was implied from the completion of the survey.

### Piloting the survey

The survey for this study was piloted among 10 colleagues of HMM and DJM, and clinicians from Aberdeen Fertility Clinic whilst waiting for ethical approval. This was deemed public involvement in research for which ethics approval is not required.^23^ Their recommendations included changing wording around sensitive questions and making clarifications around statements on the information page and changes were adopted. In preparation for the interviews, a mock interview was conducted among the research team.

#### Follow-up interviews

Follow-up interviews, based on seeking clarification of open text answers to survey questions from among respondents (‘can you tell me more about …?’), took place with volunteers either over the phone or on Skype (www.skype.com/en/), were audio recorded and lasted 10–15 minutes. The interviews were transcribed and analysed immediately, allowing data collection and analysis to build upon each other in a grounded theory fashion.^24^ In line with qualitative research conventions cited in the literature, the interview sample aimed for a total of 13–20 interviews.^25,26,27^ Participants were purposively selected for interviews based on their responses to the survey to ensure diversity in viewpoints (a smaller number of respondents who volunteered for an interview were selected and approached based on data of interest among the free-text responses, primarily to clarify disconfirming data or to elicit more detailed responses.). The last question of the survey offered respondents the opportunity to provide their email if they were interested in participating in the follow-up interview. Not all those who provided contact information were contacted. Informed consent was also taken for interviews. Potential participants were provided with an Information Sheet and Consent Form by email (see Appendices D and E). Participation was voluntary.

### Inclusion and exclusion criteria

The same inclusion criteria applied for the online survey and follow-up interviews. Those who wished to contribute to this research must have met the following inclusion criteria:

- Be 18 years of age and over;
- Be a woman;
- Have experienced current or previous menstruation.

It was not a requirement that participants had experience using FTAs. Inclusion was based on self-reported information offered by participants.

### Data analysis

#### Online survey

Data was exported to IBM SPSS Statistics (www.ibm.com/spss) Version 24 where it was coded and cleaned for analysis. Frequencies and percentages were calculated for fixed response items and free-text questions were coded and presented as themes. Categorical responses were cross tabulated in SPSS and chi-squared tests were performed to check for associations between them following generation of hypotheses from interview data.

#### Follow-up interviews

Analysis of the follow-up interview data utilised a grounded theory approach.^24^ This approach allowed for systematic and rigorous data collection with minimal preconceived ideas of what the results might produce whilst using an inductive analysis to generate theories. After each interview, the audio file was listened to and the interview was transcribed. A preliminary analysis was performed to identify emerging categories. This preliminary analysis was used to gather additional data from subsequent interviews. After all the interviews had been completed, a final analysis was carried out. Open coding of all the interview transcriptions was carried out by two persons. Interviews were analysed within and then across cases using open coding techniques to identify overall subthemes. Then a search was made for relations between the interviews and these subthemes were accepted or rejected according to new data. When reoccurring subthemes were identified, they were synthesised into four broader themes. This informed the formation of hypotheses that were tested using the quantitative data and chi-squared tests.

## Results

### Survey results

#### Sample demographics

The survey yielded 241 responses. We discarded one response so all sample demographics should be out of 240. We have doubled checked and they are all calculated out of 240. The mean age of the respondents was 29 years (range 18–61). The majority of respondents were educated to undergraduate level (*n* = 111, 46%) and were white (*n* = 220, 92%). Most of the respondents were from the UK (*n* = 195, 81%) although the survey received a global response. See Table 1 for demographic information.

**Table 1.** Demographics of the respondents.

Demographic	*n* (%)
Age group (years)	
17–24	115 (47.9)
25–36	88 (36.7)
37–42	16 (6.7)
43–48	5 (2.1)
49–54	9 (3.8)
55–61	7 (2.9)
Home country	
UK	195 (81.3)
Ireland	25 (10.4)
USA	9 (3.8)
Australia	2 (0.8)
Canada	2 (0.8)
Norway	2 (0.8)
China	1 (0.4)
Finland	1 (0.4)
Italy	1 (0.4)
South Africa	1 (0.4)
Switzerland	1 (0.4)
Education	
Secondary level	63 (26.3)
Undergraduate	111 (46.3)
Postgraduate	66 (27.5)
Ethnicity	
White	220 (91.7)
Mixed	8 (3.3)
Asian	7 (2.9)
Black	2 (0.8)
Other ethnicity/Arab	2 (0.8)
Prefer not to say	1 (0.4)
Total	240 (100.0)

#### Smartphone/tablet usage

Of the respondents, 98% (*n* = 236) have a smartphone or tablet and 94% (*n* = 227) used it to seek health information. The majority reported using their smartphone to look up health information between 1 and 2 days a week and every few weeks. Over half of the respondents (*n* = 154, 64%) reported using health apps.

#### General fertility

The following section of the survey was completed by 236 out of 241 (98%) participants. Almost a third reported that they have regular periods (*n* = 144, 61%), and 19% (*n* = 45) reported that they have irregular periods; 11% (*n* = 26) reported that they used to have periods but do not currently and 8% (*n* = 19) selected other (when asked to specify, responses were: use contraception that stops or regulates periods; currently pregnant or just had a baby; were menopausal; or had hysterectomy for irregular, heavy periods). Over half (*n* = 137, 58%) of respondents reported that they were using contraception (such as the oral contraceptive pill, the contraceptive implant, the contraceptive injection, an IUD or the barrier method), and 42% (*n* = 99) reported that they were not.

#### FTA usage

Out of the 236 respondents to this section, almost two-thirds reported that they do not use FTAs (*n* = 146, 62%) and 38% (*n* = 89) reported using them. Of the 89 women who reported using FTAs, Clue was the most popular app as 12% (*n* = 11) of respondents reported using it. The average frequency of FTA usage was between 1 and 2 days a week (*n* = 18, 20%) and every few weeks (*n* = 35, 39%). Some women also reported using the apps several times a day (*n* = 5, 6%), about once a day (*n* = 14, 16%), 3–5 days a week (*n* = 12, 14%) and less often than every few weeks (*n* = 4, 5%).

#### Experience of FTAs

From the survey data, four main motivations for the use of FTAs were:

1. To observe cycle (*n* = 63, 72%);
2. To conceive (*n* = 30, 34%);
3. To inform fertility treatment (*n* = 10, 12%);
4. As contraception (*n* = 3, 4%).

A total of 11% of respondents reported ‘other’ reason for use. Some examples of these included: to be prepared for menstruation; to track hormonal acne and mood swings; monitoring successful conception; and trying to see patterns to link to other symptoms. These reasons were not recoded to fit with our options given that participants chose to select other and provide additional text.

A number of respondents reported not sharing their data with anyone (43%, *n* = 38). Other responses were: share with partner (39%, *n* = 35); share with family and friends (10%, *n* = 9); share with healthcare professional (18%, *n* = 16); share with fertility specialist (10%, *n* = 9); and share with online community (2%, *n* = 2).

A few respondents reported using more than one app (*n* = 7) to compare the accuracy of data, to determine their consistency and to see what other apps offer.

Of the 89 respondents who reported using FTAs, 98% find the apps useful. There were 73 open text comments as to how and why the apps are useful/not useful. They were coded as in Table 2.

**Table 2.** Responses to Q15 ‘Why do you find the app useful/not useful?’ coded into themes.

‘Useful’ Themes	‘Not Useful’ Themes
Tracking helps to be prepared/plan around period	Inaccurate (usually reported in women with irregular periods)
Informative	Upsetting and cause anxiety (usually in women who are having fertility difficulties)
Tracking of other function e.g. sleep, mood and see how it correlates to cycle	Reminders are annoying
Notifications and reminders	
To maintain a history of cycles	
Easy to store, access, share data	
Predicts fertile days, can plan or avoid sex	
Community provides reassurance	

When asked if they would make any changes to the apps they are using, 30 respondents provided suggestions. These responses were coded according to emerging themes, which can be seen in Table 3.

**Table 3.** Recommended changes to FTAs coded into 12 main themes.

1	Eliminate ads
2	Add more mood choices
3	Make it easier to log data
4	More information (links to current research, relevant forums, fertility information, contraception information, general women’s health information)
5	Improved accuracy
6	Features for someone going through fertility treatment
7	Add reminders
8	Add note-taking feature
9	More specialised recommendations
10	Link to other health apps
11	Manually adjust data
12	Change design

When respondents selected ‘no’ to using FTAs they were redirected to an open text question to explain why. This question yielded 132 responses. These responses were cleaned and coded into nine themes highlighted in Table 4.

**Table 4.** Responses to Q18 ‘If you do not use FTAs, please explain why’ coded into nine themes.

1	Never knew about them
2	On contraception or not sexually active – do not see the need
3	Haven’t found one to use that I like yet
4	Menopausal or sterilised
5	Do not trust them
6	Use a diary/regular calendar
7	Apps are not user friendly/do not like them
8	Terrible with technology/do not have phone storage space
9	Too stressful/too much pressure (for women trying to get pregnant)

Of these respondents, 82% reported that they do not use other methods to track fertility. Of the 18% who reported using other methods, the majority use a paper diary but they also use temperature charts, ovulation testing kits, phone calendars and fertility monitors.

### Follow-up interview results

#### Sample

Of the 49 survey respondents who agreed to a follow-up interview and provided their email address, 16 women were contacted and invited for an interview. Eleven responded to the request. Participant codes have been assigned to protect participant identity. Table 5 gives an overview of the characteristics of the interviewees. Themes that were identified are presented in Table 6. Of the 11 interviews, there were two outliers that interested the research team. Since they did not fit into the coding frame which emerged from the majority of participant data, these interviews have been presented as two individual case studies (see Table 7).

**Table 5.** Overview of characteristics of interview participants.

ID	Age	Education Level	Country	Ethnicity	Health App User	Menstrual Status	Birth Control	Use FTAs	Why Use FTAs?	Share Data?
FTA078	23	Under-graduate	UK	White	Yes	Regular periods	IUD	Yes	Observe cycle	No
FTA193	35	Post-graduate	UK	White	Yes	Regular periods	No	Yes	Trying to conceive	No
FTA184	34	Under-graduate	UK	White	Yes	Regular periods	No	Yes	Trying to conceive	Partner
FTA060	22	Second level	UK	White	No	Irregular periods	No	Used too	n/a	n/a
FTA214	40	Under-graduate	Ireland	White	Yes	Irregular periods	NFP	Yes	Observe cycle	Doctor
FTA124	25	Under-graduate	UK	White	No	Regular periods	Contraceptive pill	No	n/a	n/a
FTA181	33	Second level	UK	White	Yes	Irregular periods	IUD	Yes	Observe cycle	No
FTA092	23	Post-graduate	UK	White	Yes	Regular periods	IUD	Yes	Observe cycle	No
FTA113	24	Second level	UK	White	Yes	Regular periods	No	Yes	Observe cycle	Doctor, family and friends
FTA051	22	Under-graduate	UK	White	Yes	Regular periods	IUD	Yes	Observe cycle	Family and friends
FTA058	22	Second level	UK	White	Yes	Regular periods	IUD	Yes	Observe cycle	No

IUD: intrauterine device; NFP: natural family planning.

**Table 6.** Subthemes and final themes that emerged from coding interviews.

Final Coding Framework	Initial Coding Framework
1. Medical grounding (science and evidence-based)	• Prediction • Contraception • Education
2. Health trackers versus non-trackers	• Early adopter and data reviewing • First timer • Phone-in-hand
3. Design (content and delivery)	• Commercialisation • Data entry and notes • ‘Pinkification’ • Additional features
4. Social and ethical aspects	• Social recommendations • Data sharing • Scepticism

**Table 7.** Interview case studies of the ‘dystopian’ versus the ‘indifferent’.

	The Dystopian Case	The Indifferent Case
Social	Used Google calendar to log previously Specific app was recommended through online forum	Did not track her cycle previously Recommended by mother to track irregular periods
Data sharing	App was recommended through online forum	Did not share data
Relationship with app and body	Developed strange relationship with body: *‘I felt like I was constantly under surveillance to a point where … I was just a bit obsessed with the fluctuations, with the accuracy of my temping, it just brought me into line with my bodily functions a little bit too closely to the point where it just wasn’t enjoyable at all’*	No comment
Stopped use	*‘It was completely disempowering!’* Deleted app after husband’s recommendation: *‘I have stopped all monitoring now’*	Kept forgetting to enter data and did not see the need for it so stopped using
Evidence-based science	*‘I do think it’s based on the 28-day model still and a lot of the things are … it’s just a really imperfect science and it makes you invest and trust it when actually I’m not the best person to be monitoring those things’*	No comment
Trust and scepticism	Would purposely enter data wrong e.g. round up temperature data *‘’cause I wanted to see my graph peak so then you think I’m not ever recording accurate data here, I want to see an ovulation and was being incredibly emotional in my use of the technology’*	Was not able to predict irregularity accurately
Emotion and attitude towards apps	Thought it was *‘so unethical and not very scientific’ ‘I think the kind of production of data on the body is problematic and I think it makes trying for a baby a lot harder.’*	Nonchalant, not bothered that it did not work for her Would laugh and refer to herself as too forgetful
Can see the benefit	*‘I imagine that if you use them and become pregnant in the start of your journey then they’re great’*	Can see why it is helpful but was not for her

### Medical grounding (science and evidence-based)

It was important to women that the apps were *‘medically sound’* (FTA078) because many use them to track other health factors. FTA214 said she uses *‘Clue because I have some ongoing medical problems that my doctor thinks is related to hormones’*. FTA184 expressed that due to a medical condition, it was important to be able to track how heavy/light her periods were as *‘It’s not the easiest thing to see in your mind’.* FTA113 said that she was diagnosed with premenstrual dysphoric disorder and to be able to diagnose it you have to diligently track your cycle *‘I did that for my doctor’s sake so I used the app’.*

#### Prediction

Accuracy of menstrual cycle prediction was very important for most of the interviewees. They rely on the apps so they could be prepared for their periods; *‘it makes it so easy so I do not get surprised’* (FTA058) or for planning purposes; *‘I suffer from heavy and painful periods so it’s to keep track so I do not schedule things when I know it’s due’* (FTA214). Those with regular cycles were generally satisfied with the accuracy of prediction of the apps that they used; *‘they seem to be able to do a really good job in being able to predict when your period is coming next’* (FTA078). However, those with irregular cycles expressed frustration that their app was not individualised to their own cycle; *‘what I’d quite like it to do is … to work out some sort of prediction rather than just go off a block of 28 days’* (FTA181). Women also expressed an interest in learning how the prediction methods work to see if they were personalised based on their own data or whether they were just based on a generic average cycle prediction; *‘it would be interesting to see how their prediction methods work. I wonder if it’s based on my last entry or a series of entries that they predict my next one’* (FTA051).

#### Contraception

Of the 11 interviewees, two expressed an interest in using FTAs as a form of contraception; ‘*I’ve never really tried that form of contraception but I’d be interested in trying that at some point’* (FTA078). FTA124 said she doesn’t currently use FTAs but expressed interest in coming off the oral contraceptive pill and switching to using Natural Cycles as a form of contraception; *‘ It’s safe, from what I’ve read, it’s pretty accurate and I do not see why not to give it a try’* (FTA124).

In contrast, FTA113 expressed concern over using an app as a form of contraception because she knows people who got pregnant whilst using Natural Cycles as contraception; *‘I have two friends who have become pregnant using it even though they were really careful …  So I obviously have a really negative view of that as a method’.*

#### Education

Many women found the apps educational and admitted that this was the first time they have truly understood their own bodies. FTA078 expressed *‘I think I’ve learned more about my own reproductive system through this app than anything else’* as did FTA113; *‘I didn’t know anything about my cycle before I started using the app’*. One woman reported that *‘since using Clue I’ve realised I have ovulation pain’* (FTA058). The use of the apps is allowing women to better understand each stage of their cycle and to find correlations between their cycle and symptoms.

### Health trackers versus non-trackers

#### Early adopter and data reviewing

Three interviewees reported using FTAs long-term; *‘I’ve used menstrual calendar for about 9 years’* (FTA184). FTA181 said she’s used MyCalendar for *‘about 4 or 5 years’.* FTA193 said that she has had *‘a few others … going back to like 2012’.* Interestingly, the three women who were early adopters of the apps all reported that they like to go back and review their data; *‘It’s reassuring that I can just flick back and see it’* (FTA184). FTA184 stated that she could go back to her data when she previously became pregnant; *‘I like to compare it’.*

#### First timer

Of the 11 interviewees, six reported that using FTAs was their first time tracking their periods; *‘using Clue is the first time I’ve ever tracked before’* (FTA214). A lot of women reported that they were first-time trackers.

#### Phone-in-hand

It emerged that women like to use apps on their phones to record their menstrual cycle data because it is convenient; *‘my phone is in my hand 24 hours a day so I said I’d see what apps are available’* (FTA214). Accessibility was another feature that made tracking on a smartphone desirable; *‘I like having it in my pocket wherever I am’* (FTA051).

### Design (content and delivery)

#### Commercialisation

Three women discussed advertisements in their interviews. FTA113 didn’t seem too concerned that the apps she uses *‘has some ads’*; however, FTA051 stated that she *‘had a different app before but it had pop up ads’* which she disliked and made her change to using a different app. FTA078 liked that Clue did not *‘try to get you to buy any extras’*. There was a trend in women who were using the app to observe their cycle to keep their data private and inconspicuous; *‘it’s more discreet to have Clue that just looks like a health app … I like that it sits next to my Apple Health and is a similar design … like kind of medical … makes me feel like I’m just tracking my health’* (FTA051). One women expressed concern over the designers of these apps not listening to the recommendations of users; *‘I just wonder what kind of patient-public interaction there is with the designers’* (FTA214).

#### Data entry and notes

Generally, women were very positive about the ease of entering data into their app; *‘if you forget to track it’s easy to go back and put that data in’* (FTA078). Adding notes was a key feature that women found important when the standard format of the app did not allow them to input all data that they felt was relevant; *‘I add notes like there’s no tomorrow’* (FTA184). Interestingly the two women who were using apps to try to conceive were the only women who used the apps extremely diligently. The other women had a more nonchalant approach to entering data often reporting that they would forget to enter data; *‘I do not use it religiously, I try to put in my dates and sometimes add in notes’* (FTA181).

#### ‘Pinkification’ (i.e. the use of traditionally feminine design features)

Two women commented on the gendered design of most FTAs. FTA092 commented that *‘I chose Clue because it’s the only app that wasn’t pink’.* FTA051 also found the gendered design of her previous app insulting*; ‘my last app had a pink flower and was called MyDays or something … I felt like they were trying to lure me in with this kind of “women’s” approach’* (FTA051). She subsequently stopped using that app and downloaded Clue.

#### Additional features

Some FTAs have features to track other determinants of health such as diet, exercise, mood, sleep and sexual activity. Six women mentioned that they liked the additional tracking options that their apps offered. FTA051 said *‘you can track your hair, skin, diet, exercise … all those lifestyle things … I can look at Clue and see when I’ve been drinking or eating lots of bad food … I appreciate the scope that Clue offers’.* Similarly FTA184 said that *‘tracking my weight is a big thing for me’*. Some women often found that their cycle is correlated with their mood; *‘my mood is very dependent on my cycle … I use it to track that to understand myself better’* (FTA113). FTA058 recognised the benefit of tracking other aspects of health; *‘you can track loads of stuff on it but also you do not have to’*. She liked that her app didn’t pester her with *‘a pop up of like “do not forget to track how much water you’ve drank today”’* and appreciated that you had the option to track lots of bodily functions or simply *‘what days you are on your period which is nice’.*

### Social and ethical aspects

These aspects of using FTAs remain poorly understood and under-regulated. Women are beginning to discuss if and how FTAs share their data. Most women were unaware of how their data was being utilised by the app owners and were unbothered *‘if anyone is desperate to know when my periods are then I really do not care’* (FTA181). Only one woman expressed suspicion about her data privacy and admitted to registering with an alternative email address. She also claimed that *‘I do not put in my exact date of birth, I put in the right year but swap month and date, put in my middle name and married name … I think I’m being very clever’* (FTA214).

#### Social recommendations

Two women reported that their mother had recommended that they start tracking but not specifically via an app. Two others said that the app Clue was recommended by a friend. One woman came across an app recommended on an online forum and another woman saw Natural Cycles advertised on social media. The remaining women discovered the apps on their own after a recommendation from their doctor to start tracking their cycles. This is evidence of the fact that discussing periods is becoming less of a taboo and women are sharing their tracking practices in their social circles both in person and online.

#### Data sharing

The majority of women reported tracking their cycles so they could share their data with a healthcare professional or to have as a precautionary measure; *‘It’s definitely helpful for doctor’s appointments’* (FTA078) and FTA058 said *‘If my gynaecologist needed to know anything I have it just in case’*. One survey respondent said that she is not currently sharing her data but she *‘intends to use it to start a conversation with her doctor’* (FTA214)*.* Interestingly, FTA184 said that she is not taken as seriously by her doctor unless she has her app data as proof; *‘I can go to my doctor and it’s nice to be solid on the data  … You tend to get ignored if you do not have data to back yourself up’*.

Women reported sharing data with family and friends but in a casual way that does not involve showing them specific details of the app; *‘It’s helpful to give family and friend a heads up to know if I’ll be a bit sad or down’* (FTA113) and to compare their app and data tracking with others; *‘It’s helpful to see how other women are tracking their cycle’* (FTA051).

#### Scepticism

The two women trying to conceive expressed the most scepticism about the accuracy and reliability of the apps; *‘It’s just a really imperfect science … so unethical and not very scientific’* (FTA193) and FTA184 says *‘I do not trust Yono … I do not think they’ve found a way what they’ve decided is your Basal Body Temperature (BBT) from the ear results … I trust mouth temperature more’*.

### Interview case studies

There were two outliers that did not fit into the coding frame which emerged from the majority of participant data; these interviews have been presented as two individual case studies (see Table 7). They are the only two interviewees who reported giving up using the apps, but for very different reasons. They have been identified as the dystopian case and the indifferent case. The former is an example of someone who has been obsessed by and consumed with tracking her fertility to the point where she found it unethical, unscientific and detrimental to her health. The latter found the app unhelpful because she simply forgot to update it and seemed unaffected by the fact that the app did not suit her needs.

#### Hypotheses testing

Once the interview data was analysed and themes began to emerge the researchers formed four hypotheses and tested them in the quantitative survey data (see Table 8). Hypothesis, null hypothesis, result and explanation are all reported. To better understand why women were/were not using FTAs, FTA usage was cross tabulated with health app usage, birth control usage, age group and menstrual status.

**Table 8.** Hypotheses that were formed after analysis of follow-up interview data.

Hypothesis 1	Women who use health apps are more likely to use FTAs than women who do not use health apps
Null Hypothesis 1	There is no association between FTA usage and health app usage
Result 1	Reject null hypothesis (χ^2 ^= 24.153, df = 1, *p* = 0.001)
Explanation 1	Out of 154 women who use health apps, 76 also use FTAs (49%). Out of 82 women who do not use health apps, 13 use FTAs (16%). This difference was significant (χ^2 ^= 24.153, df = 1, *p* = 0.001).
Hypothesis 2	Women using contraception are less likely to use FTAs
Null Hypothesis 2	Birth control use does not affect FTA use
Result 2	Reject null hypothesis (χ^2 ^= 9.897, df = 1, *p* = 0.002)
Explanation 2	Of the women using birth control, 40 (29%) reported using FTAs. Of the women that do not use birth control, 49 (50%) used FTAs. This association was significant (χ^2 ^= 9.897, df = 1, *p* = 0.002). Perhaps this is because many types of birth control regulate the menstrual cycle so a woman using birth control may not feel the need to track.
Hypothesis 3	Younger women are more likely to use FTAs than older women
Null Hypothesis 3	There is no relationship between FTA usage and age
Result 3	Reject null hypothesis (χ^2 ^= 27.206, df = 5, *p* = 0.001)
Explanation 3	Of the women aged 18–24, 28% used FTAs; of women aged 25–32, 45% used FTAs; of women aged 33–39, 67% used FTAs; of women over 40, 20% used FTAs. This trend shows that as age increases, FTA usage increases until the age of 40 when it decreases. This relationship is significant (χ^2 ^= 27.206, df = 5, *p* = 0.001).
Hypothesis 4	Women with regular menstrual cycles are more likely to use FTAs that women with irregular cycles
Null Hypothesis 4	There is no relationship between FTA usage and menstrual status
Result 4	Accept null hypothesis (χ^2 ^= 5.293, df = 4, *p* = 0.259)
Explanation 4	There is no association between the use of FTAs and menstrual status, e.g. regular/irregular periods. Of the women who have regular periods, 60 (42%) reported using FTAs. Of the women who had irregular periods, 40% reported using FTAs. The difference was not significant (χ^2 ^= 5.293, df = 4, *p* = 0.259).

## Discussion

### Summary of findings

This study produced novel findings on women’s uses of and relationships with FTAs. The survey was only live for five days, but received a good response (*n* = 241). Volunteers for interviews were forthcoming, perhaps because more women are beginning to use FTAs and want to share their experiences and views.

Four main uses of FTAs were identified: to observe; to conceive; to inform fertility treatment; and as contraception. Women suggested many changes to the apps they use. This indicates that there are a range of motivations for use and that needs vary greatly between users.

It is important to note that 82% of women who use FTAs reported that they had not previously used another form of fertility tracking. Findings comparable with ours were reported from a survey study of 310 women in 2015, although the survey was conducted face to face and specifically among women seeking fertility treatment.^28^ Conversely, it found that younger women who are trying to become pregnant and who have regular cycles are more likely than other women to use smartphone apps. Further data was obtained from a large-scale US study of over one million women who use fertility tracking smartphone apps, although the relationship between tracking and motivation for use is unclear from the published abstract.^29^

#### Follow-up interviews

The follow-up interviews provided deeper insights. The themes that were identified highlighted that women seek FTAs with more accurate prediction methods and more information. Women felt FTAs offer the best method to track fertility because of the large amount of data they can store and the convenient accessibility that smartphones provide. There was a level of dissatisfaction when it came to design. Most women had suggestions about how to improve both the content and delivery of these apps.

The two case studies reinforced the finding that users have varying motivations and needs which could be met if apps were more customisable to the individual. Lupton^2^ claimed that health apps can empower users to monitor and manage their own health but this is clearly not the case for all users.

### Hypotheses

#### Hypothesis 1

Women who use health apps are more likely to use FTAs than women who do not use health apps. Self-tracking is an interlinked community and often if you track one determinant of health, you are likely to track others. It is still unknown whether women are using health apps as a gateway to fertility tracking or vice versa.

#### Hypothesis 2

Among women who use contraception, the majority are less likely to use FTAs than women who do not use contraception. This may be because many types of birth control regulate the menstrual cycle so a woman using birth control may not feel the need to track.

#### Hypothesis 3

Women use FTAs more up until 40 years of age, when its use decreases. This may be because younger women engage more with technology and use dips as perceived childbearing age decreases. FTAs must not be dismissed for older women as they could also be useful to track fertility in later onset treatment or to record changes as a woman goes through menopause.

#### Hypothesis 4

There was no association found between the use of FTAs and menstrual status, e.g. regular/irregular periods. Women with irregular cycles are still using FTAs even though they feel like they do not meet their needs. This further demonstrates that women with varying motivations are still using FTAs and their needs should be met.

### Implications of findings

Women’s uses of and relationships with FTAs have implications for various stakeholders, introduced below. Collaboration between stakeholders is necessary to satisfy user needs. Some extensions of our analyses and speculations as to how are presented.

#### Implications for reproductive health services

Healthcare professionals providing care and support for women need to take women’s app use into account and recognise both the potential and limitations of these apps. Tracking via apps has helped women in this study notice menstrual irregularities that they have then discussed with their doctors. Recommendations to health care providers to guide their support of FTA users has been developed.^16^ Calls for users to be supported appropriately^16^ to avoid their health and wellbeing being compromised^30^ have been made. One nursing practice study highlights the need for nurses to explore available apps, incorporate their use into practice and share insights with peers to develop practical knowledge.^31^ This is not just for fertility awareness, but for reproductive health more broadly.

#### Implications for regulators

A concern with commercial apps is that they are not regulated or approved for use by official bodies (with the exception of Natural Cycles), but consumers may erroneously assume that they are. There are hundreds of FTAs available and they vary widely in their prediction methods and accuracy. There are ethical and privacy issues surrounding the data they collect. For example, commercial apps could sell users’ data to third parties, including employers, which may have implications for menstrual leave and/or maternity leave.

#### Implications for developers

Smartphones are now an extension of our bodies. Our relationship with technology is obsessive and we are generating/consuming more data than ever before. This study found that many women do not track their fertility using an app. With the rising use of health apps, developers might benefit from keeping their audiences’ needs in mind, or consulting them when designing FTAs. Services might also consider partnering with providers of apps or designing their own apps to ensure a two-way flow of evidence and information can improve apps and knowledge.

App developers should provide the most accurate prediction methods possible. There are many external factors that can affect a woman’s cycle and developers should take these into consideration and allow women to manually adjust data. FTAs should be designed for customisation. When setting them up, a woman should be asked more specific questions with a wider range of options about how she would like her app to be designed, what level of extra features she would like, what type of user she is and so on. This recommendation that apps must be aimed at a wider audience is in line with Lupton’s recommendations ‘that further research/consideration is needed for marginalised groups such as lesbian, gay, bisexual or transgender (LGBT) people, or differing socio-economic backgrounds’ regarding pregnancy apps and health apps in general.^13,32^ Developers should also consider creating professional and gender-neutral app layouts to support the various types of user, e.g. not pink and flowery.

### Women’s needs

Women in this study expressed interest in tracking symptoms, such as mood, skin, diet and exercise. Perhaps they are using FTAs as a gateway to tracking other determinants of health. If this is the case then there should be better interoperability between various health apps so data can be collected and transferred easily.

Compared with other determinants of health, e.g. lifestyle choices, fertility tracking is very different. Women do not have any control over their menstrual cycles. However, the importance of simply tracking to observe, rather than to motivate behaviour change/influence outcomes, must not be overlooked. Observing can help women to understand their symptoms and sometimes explain causes and lead to diagnosis.

Many participants reported that using FTAs were informative and educational. Women are only formally taught about their reproductive health as children when it can be an uncomfortable topic to discuss. FTAs give women the opportunity to learn about their own bodies in a safe environment. If FTAs are meeting the educational needs of so many women then perhaps some apps should have a feature for younger prepubescent women who may want to educate themselves in a private and safe environment. Graphics and colours are considered to be important and although our study found that ‘pinkification’ was not attractive, another study referred to ‘pink and lovely graphics’ as appealing.^31^

### Limitations of study

The main limitation of this study is that the sample size of the survey portion of the study is very small, even ignoring the fact that only 89 of the 241 respondents actually used an FTA. In addition, we cannot be sure that women’s attitudes to FTAs can be disentangled from their view of their effectiveness.

Furthermore, this study used a convenience sample so the findings are not generalisable for all consumers of FTAs. It is also recognised that, in grounded theory, sampling is not demographically representative. The demographic makeup was heavily influenced by the research team’s social media networks. This survey’s sample was homogeneous: the majority were white, from the UK and educated to undergraduate level. In a future study it would be interesting to see if there were differing results across different ages, education levels, economic statuses, cultural groups and ethnic groups.

Receiving ethical approval took longer than expected due to availability of board members (five as opposed to the average length of two weeks). This limited the amount of time that the online survey was live: for only five days. If it had been available online for longer, it might have provided a larger and more demographically diverse sample.

Selecting the term ‘fertility tracking’ app as opposed to ‘period tracking’ or ‘menstrual cycle tracking’ may have had an effect on the number of women who participated in the study. Women who track with the aim to observe their cycle, and who are not seeking to become pregnant, may not identify with the word fertility and may not feel it is applicable to them.

### Strengths of study

By collecting complementary quantitative and qualitative data, breadth and depth of understanding were elicited. Mixed methods research provides a more complete and comprehensive account of the research problem than either quantitative or qualitative approaches alone. A mixed methods paradigm can strengthen the quality of the research by combining the strengths of each approach and mitigating the internal limitations of each.^33^

When writing up the results, the researchers discovered a similar study conducted by Epstein et al.^34^ in the USA. Although this study differs in objectives and methodology, these findings mirror many of their findings, suggesting there is a level of consistency in women’s relationships with and uses of FTAs.

### Reflexivity and research team potential biases

It is important to note that the primary researcher is using a form of contraception that has affected her menstrual cycle and has stopped her periods. Therefore, she does not use any FTAs and has no preference towards a particular app. Although she noticed a trend towards Clue being the favourite app, she was able to remain unbiased. The other female member of the research team uses no hormonal contraception, but is an early adopter and tech enthusiast and uses various apps, including FTAs, for research purposes. Her regular app is Clue and she has a paid subscription to Natural Cycles. She has also tracked her menstrual cycles using paper since first menstruating and continues to do so.

### Recommendations for future research

Future research should explore women’s uses of FTAs across education level, economic status and cultural and ethnic groups.

This study demonstrated that there are a range of user groups who feel that their needs are not being met by currently available FTAs. Most apps are designed for narrow user groups, women who are healthy; are sexually active; are heterosexual; have regular periods; and have no fertility issues. Future research should consider the needs of individuals who are:

- Infertile or struggling to conceive;
- Of non-normative gender identity;
- Experiencing other health concerns that impact menstruation;
- Any sexuality other than heterosexual;
- Experiencing irregular periods.

Further research into the views of consumers of these technologies can contribute to understandings of how to create more user-friendly products that can benefit health and social care providers and consumers.

## Conclusion

App developers and health services hoping to develop clinically endorsed apps need to listen more to the voices of their users and take their suggestions on board when developing future FTAs to improve user experience. Social and ethical aspects of FTAs are still poorly understood and under-regulated. However, with increasing use of FTAs^7^ and a general shift to providing evidence-based healthcare via technology, there will be increased onus on regulatory bodies.

This research emphasises the importance of FTAs being personalised to the individual to support the differing motivations of users. It recommended that app developers design for accuracy, individualisation and inclusion. Collaboration between various stakeholders is also necessary to satisfy user needs. Future research should focus on further exploring the dynamic relationships between women and FTAs to inform health services, regulators and app developers, and therefore improve the experience for users of various menstrual and/or birth control statuses and to support the integration of FTAs into health care.

## Supplemental Material

Appendix A to D -Supplemental material for A mixed methods exploratory study of women’s relationships with and uses of fertility tracking apps Click here for additional data file. Supplemental material, Appendix A to D for A mixed methods exploratory study of women’s relationships with and uses of fertility tracking apps by Katie Gambier-Ross, David J McLernon and Heather M Morgan in Digital Health
