# Supplementary material for: A mixed methods exploratory study of women’s relationships with and uses of fertility tracking apps
Source: Digit Health. 2018 Jul 25;4:2055207618785077. doi: 10.1177/2055207618785077 (PMC6136106; doi:10.1177/2055207618785077)
Supplement: Appendix A to D -Supplemental material for A mixed methods exploratory study of women’s relationships with and uses of fertility tracking apps [file Appendix_A_D.pdf]

Appendix A-Methodological Design

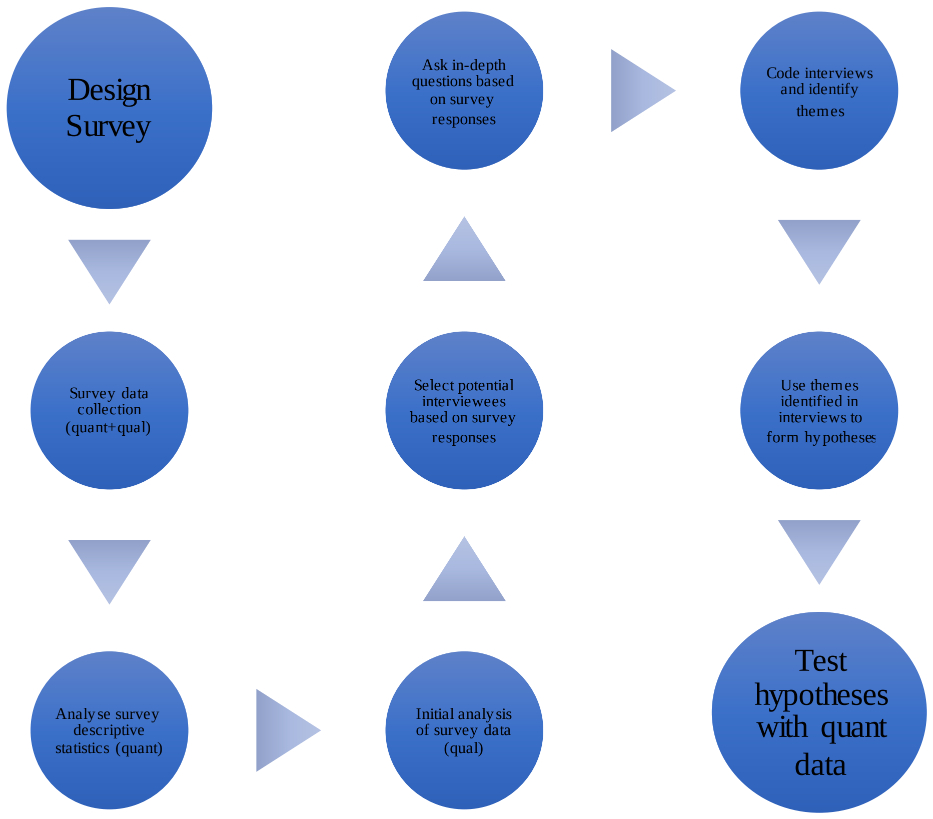

1 **Appendix B-Study Protocol**

STUDY PROTOCOL

Draft Title:	**An exploratory study of women using fertility tracking apps**
Sponsor:	University of Aberdeen
Funder:	School of Medicine, Medical Science and Nutrition, University of Aberdeen
Researcher:	Katie Gambier-Ross
First supervisor:	Dr Heather Morgan
Second supervisor:	Dr David McLernon

1. INTRODUCTION

1.1. Background

Digital technologies are transforming the way we live. They dominate our working and social everyday lives, so it is no surprise that these technologies have rooted themselves in health sectors as well. It is an exciting prospect that digital technologies are making health and social care more convenient, more coordinated and more affordable. In recent years, there has been an increased interest in self-management of health. Health policies internationally advocate 'support for self-management', but it is not clear how the promise of the concept can be fulfilled (Morgan *et al.*, 2016). This interest has accelerated even further in recent months with the rise of affordable and portable technologies that allow users quantify and measure their health. This has led to an increase in the popularity of movements that encourage users to collect their health information as a means of self-surveillance and self-discipline (Lupton, 2014). A fast-emerging aspect of the quantified self-movement is fertility tracking (Caddy, 2015). Apart from "activity trackers" fertility trackers are the most frequently downloaded kind of health app in the Apple Store (Weigel, 2016).

Although fertility tracking apps are a relatively new concept, the act of fertility tracking itself is not. Menstrual cycle tracking originates from the medical field and had its roots long before the age of quantified self apps. Since the early 1930s, women have been using calendar based or fertility awareness based contraception methods (Singer, 2004). Usually women who were using one of those methods documented their symptoms with pen and paper. With the rise of mobile technology and increasing smartphone use, period tracking habits have experienced almost a complete

shift from using pen-and-paper to using mobile apps. However, little research has been done on how women are using these apps and what the implications of that might be.

It has previously been argued that no calendar method is completely effective (Lamprecht and Grummer-Strawn, 1996). Conversely, a recent study found that correct use of the fertility awareness-based mobile app NaturalCycles can be an effective method of contraception (Berglund Scherwitzl *et al.*, 2016). Further fertility awareness-based methods have been developed that take changes in one or more of the primary fertility signs such as basal body temperature, cervical mucus or cervical position of women into account. These methods have both advantages and disadvantages. Most methods entail no or minimal cost, no drugs or physical devices are required, and medical personnel are not required. On the other hand, signs and symptoms of the fertile phase may be subtle (IPPF, 1995) and irregular cycles can make these methods difficult. It is also important to note, given that several apps offer these tracking options, that the comparative efficacy of fertility awareness-based methods of contraception remains unknown (Grimes *et al.*, 2004). The findings on the effectiveness of fertility awareness-based methods vary greatly in the literature depending on the fertility indicators used (menstruation, temperature, cervical mucus, or a combination), the social settings (industrialised or developing countries) and the study design itself (Berglund Scherwitzl *et al.*, 2016).

In this study, fertility tracker apps are defined as; a smartphone or tablet application that allows users to record the dates of their periods, as well as additional information, such as mood, symptoms, sex, and medications. It can predict fertility using an ovulation calculator based on the information entered and can help women learn about their fertility, whether avoiding or attempting pregnancy. It might also assist those undergoing fertility treatments.

1.2. Rationale for Study

It is important to improve our understandings of the relationships between health and digital technologies. By gathering, sharing and analysing large amounts of data these fertility tracking apps are revolutionising reproductive medicine (Weigel, 2016). They may enable new insights into long understudied aspects of how female bodies are experienced ad understood. Fertility trackers can clearly improve women's abilities to manage information and to communicate with their doctors and partners and significant others. They may help to shift taboo around periods and to create spaces online for discussing common problems such as endometriosis or fertility problems. This study aims to provide an initial probe in exploring how women are using fertility tracking apps and what their perceptions of these apps are. Hopefully this exploratory research will identify themes so future wok can be conducted in this field.

2. STUDY OBJECTIVES

- To explore women's use of fertility tracking apps
- To identify themes e.g. why do people use them?, what do participants find useful/not useful about the apps? Do participants share their data? Would participants make any changes to the apps? Etc.
- To be a platform for further research

3. STUDY DESIGN

3.1. Study Description

The researcher will employ a mixed methods approach, involving collection, analysis and synthesis of data obtained through an online questionnaire and follow up interviews.
The questionnaire for this study will be piloted amongst a few colleagues in the HSRU and Aberdeen Fertility Clinic and the Fertility Network whilst waiting for ethical approval. If they have any recommendations to change the questionnaire, then changes will be made with the approval of CERB.

4. STUDY POPULATION

4.1. Number of Participants

There is no upper limit to the number of responses that the online questionnaire will receive.

The semi-structured interview sample will aim for a total of 13 to 20 interviews either on the phone or by Skype. Participant will be purposively selected for interviews based on their responses to the survey to ensure diversity in viewpoints.

4.2. Inclusion Criteria

Participants must meet the following criteria;
- Be over the age of 18
- Be a woman
- Experienced current or previous menstruation

4.3. Exclusion Criteria

Children (under 18 years) will be excluded from this study. Although the researcher recognises that those under 18 may be using these apps, interviewing minors may need parental approval, supervisor and extra training. The researcher does not feel qualified to undertake this.

5. PARTICIPANT SELECTION AND ENROLMENT

5.1. **Consenting Participants**

Informed consent will be taken for questionnaires – information will be provided on the front page of the online questionnaire along with the statement; "It is important that you understand that by completing and submitting the questionnaire that you are consenting to participate in the study."

Informed consent will be taken for interviews. Potential participants will be provided with a Participant Information Sheet and Consent Form (by email) and will have 24 hours to consider whether they would like to participate in the research. There will be no obligation to participate in the research. Should participants wish to take part, the researcher will go through a consent form with the participants at the beginning of the interview to establish and record vocal consent. The researcher will sign the consent form on behalf of the participant.

5.2. **Screening for Eligibility**

Men and individuals under 18 years old will not be eligible to participate in this research.

5.3. **Ineligible and Non-Recruited Participants**

Any participant will be eligible for an interview. Interviewees will be adult (over 18) volunteers.

6. RANDOMISATION AND BLINDING

6.1. **Randomisation Details**

Not applicable.

6.2. **Blinding**

Not applicable.

6.3. **Withdrawal Procedures**

Participants will be free to withdraw at any time and without giving a reason. A decision to withdraw at any time, or a decision not to take part, will not affect the standard of care people receive or any legal, health care or employment rights.

If a participant who has given informed consent loses capacity to consent during the study, the participant would be withdrawn from the study. Identifiable data already collected with consent would be retained and used in the study. No further data would be collected or any other research procedures carried out on or in relation to the participant.

7. DATA COLLECTION AND MANAGEMENT

7.1. **Data Collection**

The online questionnaire will capture demographic and fertility app usage data. It will consist of both closed and open questions. For the interviews, primary data will be collected in the form of notes, audio files and transcriptions and these will be collected and stored as research data. Audio recording devices will be used to capture interview data and to enable interviews to be transcribed verbatim for subsequent analysis.

During the interviews, if participants are struggling to discuss sensitive and potentially distressing issues, the interviewer will allow the participant to pause, move on from that topic or end the interview at any point.

Research data will be stored securely on password protected University of Aberdeen computers to enable analysis with only members of the direct research team having access. Personal computers/laptops with University compliant security settings may also be used when off site. Paper copies will be held in locked units at the University of Aberdeen that only the research team have access to. Although we will not collect personal data, any identifying details for a participant will be stored securely and separately from their interview data following the University of Aberdeen Research Governance Guidelines (http://www.abdn.ac.uk/staffnet/documents/policy-zone-research-and-knowledge-exchange/Res_Gov_Handbook_-_June_2015.pdf).

Identifiable materials from all participants will be stored separately to any transcripts or completed questionnaires. Identifiable participant information will be kept on a separate spread sheet or log and used for linking to anonymised data. This spread sheet or log will be restricted and only accessible by the researchers.

Anonymised data will be archived per University of Aberdeen guidelines. Access will only be for research purposes. Direct quotes may be used in the publication of research findings, but these will not be attributed to named individuals and any identifiable information will be removed.

7.2. Data Management System

Any identifiable data will be stored securely on password protected devices and systems and paper copies will be locked securely in tambours at the University of Aberdeen.

The data generated by the study will be analysed at the University of Aberdeen by Katie Gambier-Ross, Dr Heather Morgan and Dr David McLernon.

The data will be stored and subsequently archived on the secure networked PCs at the University of Aberdeen and paper copies will be stored in a locked filing cabinet in the Health Services Research Unit at the University of Aberdeen.

8. STATISTICS AND DATA ANALYSIS

8.1. Sample Size Calculation

No formal sample size calculation was performed as there is no upper limit on for the questionnaire and this is predominantly exploratory qualitative research study. There is no upper limit to the number of responses to the questionnaire and we would like to interview a maximum of 20 participants.

8.2. Proposed Analysis

Questionnaire data will be recorded in SPSS and coded and cleaned for analysis. Closed questions will be presented as frequencies and percentages. Analysis of open questions and follow up interview data will involve an iterative process, which will draw on a grounded theory approach. This approach allows for systematic and rigorous data collection while using an inductive analysis to systematically and rigorously generate theory.

8.3. Missing Data

If there is missing data in the questionnaire, we will quantify the missing data for responses of each question.

8.4. Transfer of Data

Not applicable.

9. TRIAL/STUDY MANAGEMENT AND OVERSIGHT ARRANGEMENTS

9.1. Trial/Study Management

Dr. Heather Morgan, Research Fellow, Health Services Research Unit, will oversee the study, in conjunction with Dr. David McLernon, Research Fellow, Medical Statistics.

10. GOOD CLINICAL PRACTICE

10.1. Ethical Conduct of the Study

The study will be conducted in accordance with the protocol approved by the College Ethics Review Board (CERB) and in line with the principles of Good Clinical Practice and HSRU's research governance framework http://www.abdn.ac.uk/hsru/research/quality/documents/governance/.

10.1.1. Confidentiality

All documentation will be handled in a manner designed to maintain participant confidentiality. No identifiable information/contact details will be kept alongside data and data will be anonymized. All records will be kept in a

secure storage area with limited access to study staff only. The researchers will not disclose or use for any purpose other than performance of the study.

10.1.2. Data Protection

The researchers involved with this study will comply with the requirements of the Data Protection Act 1998 with regard to the collection, storage, processing and disclosure of personal information and will uphold the Act's core principles. Access to collated participant data will be restricted to the researchers.

Computers used to collate the data will have limited access measures via user names and passwords.

Published results will not contain any personal data that could allow identification of individual participants.

11. STUDY CONDUCT RESPONSIBILITIES

11.1. Protocol Amendments, Deviations and Breaches

The researcher will seek approval for any amendments to the Protocol or other study documents from CERB. Amendments to the protocol or other study docs will not be implemented without these approvals.

11.2. Study Record Retention

Archiving of study documents will take place after the study ends.

The data will be stored for up to ten years and subsequently archived on the secure networked PCs at the University of Aberdeen and paper copies will be stored in a locked filing cabinet in the Health Services Research Unit at the University of Aberdeen.

11.3. End of Study

The end of study is defined as 31 March 2017, when the study will end and the dissertation will be submitted as part of University examination.

12. REPORTING, PUBLICATIONS AND NOTIFICATION OF RESULTS

12.1. Authorship Policy

Ownership of the data arising from this study resides with the study team and the University of Aberdeen. On completion of the study, the study data will be analysed and written up.

12.2. Publication

This research will be used for publication and presentation at scientific meetings. Investigators have the right to publish orally or in writing the results of the study.

Summaries of results will also be offered to participants.

12.3. Peer Review

The protocol will be reviewed by CERB.

REFERENCES

Berglund Scherwitzl, E., Gemzell Danielsson, K., Sellberg, J. and Scherwitzl, R. (2016). Fertility awareness-based mobile application for contraception. *The European Journal of Contraception & Reproductive Health Care*, 21:3, 234-241, DOI: 10.3109/13625187.2016.1154143.

Caddy, B. (2015). *The Quantified Woman: Fertility tracking and the future of our reproductive health*. [online] Wareable. Available at: https://www.wareable.com/health-and-wellbeing/the-quantified-woman-fertility-tracking-1914 [Accessed 30 Jan. 2017].

Grimes DA, Gallo MF, Halpern V, Nanda K, Schulz KF, Lopez LM. Fertility awareness-based methods for contraception. *Cochrane Database of Systematic Reviews* 2004, Issue 4. Art. No.: CD004860. DOI: 10.1002/14651858.CD004860.pub2.

International Planned Parenthood Federation. (1995). Statement on periodic abstinence for family planning. London: International Planned Parenthood Federation, 1995.

Lamprecht, V. and Grummer-Strawn, L. (1996). Development of new formulas to identify the fertile time of the menstrual cycle. *Contraception*, 54(6), pp.339-343.

Lupton, D. (2014). Quantified sex: A critical analysis of sexual and reproductive self-tracking using apps. *Culture, Health, and Society, 17*(4), 440-453.

Morgan, H., Entwistle, V., Cribb, A., Christmas, S., Owens, J., Skea, Z. and Watt, I. (2016). We need to talk about purpose: a critical interpretive synthesis of health and social care professionals' approaches to self-management support for people with long-term conditions. *Health Expectations*.

Singer, K. (2004). The garden of fertility: A guide to charting your fertility signals to prevent or achieve pregnancy—naturally—and to gauge your reproductive health. New York: Penguin Group (USA) Inc.

Weigel, M. (2016). *'Fitbit for your period': the rise of fertility tracking | Moira Weigel.* [online] the Guardian. Available at: https://www.theguardian.com/technology/2016/mar/23/fitbit-for-your-period-the-rise-of-fertility-tracking [Accessed 30 Jan. 2017].

Wilcox, A., Dunson, D. and Baird, D. (2000). The timing of the "fertile window" in the menstrual cycle: day specific estimates from a prospective study. *BMJ*, 321(7271), pp.1259-1262.

Wilcox, A., Weinberg, C. and Baird, D. (1995). Timing of Sexual Intercourse in Relation to Ovulation — Effects on the Probability of Conception, Survival of the Pregnancy, and Sex of the Baby. *New England Journal of Medicine*, 333(23), pp.1517-1521.

Appendix C-Online Survey

1. Welcome to My Survey

My name is Katie Gambier-Ross and I am a final year student at University of Aberdeen. As part of my undergraduate dissertation, I will be exploring how women use fertility trackers. This research is being conducted as part of my studies and will be submitted for examination.

Women are invited to participate in this study by completing the following anonymous questionnaire. This questionnaire is comprised of 20 questions and will only take 12-15 minutes of your time to complete. Participation is voluntary.

In recent years, there has been an increased interest in self-management of health. A fast-emerging aspect of the quantified self-movement is fertility tracking. With the rise of mobile technology, fertility tracking habits are done almost exclusively on mobile phone apps. However, little research has been done on how women are using these apps and what the implications of that might be.

In this study, fertility tracker apps are defined as; a smartphone or tablet application that allows users to record the dates of their periods, as well as additional information, such as mood, symptoms, sex, and medications. It can predict fertility using an ovulation calculator based on the information entered and can help women learn about their fertility, whether avoiding or attempting pregnancy. It might also assist those undergoing fertility treatments.

Participants should be over 18 years of age and should experience current or previous menstruation.

It is important that you understand that by completing and submitting the questionnaire that you are consenting to participate in the study.

If you have any queries about this research you can contact me at fertilitytrackerresearch.abdn@gmail.com. Alternatively, you can contact my dissertation supervisor Dr. Heather Morgan at h.morgan@abdn.ac.uk.

Thank you!

2. Demographics

* 1. What is your age?

* 2. What is the highest level of education you have completed?

* 3. In what country do you live?

1

* 4. Please describe your ethnicity.

- ◯ White- English/ Welsh/ Scottish/ Northern Irish/ British
- ◯ White- Irish
- ◯ White- Gypsy or Irish Traveller
- ◯ White- Any other white background
- ◯ Mixed- White and Black Caribbean
- ◯ Mixed- White and Black African
- ◯ Mixed- White and Asian
- ◯ Mixed- Other mixed/mutiple ethnic background
- ◯ Asian/Asian British- Indian
- ◯ Asian/Asian British- Pakistani
- ◯ Asian/Asian British- Bangladeshi
- ◯ Asian/Asian British- Chinese
- ◯ Asian/Asian British- Any other Asian background
- ◯ Black- African
- ◯ Black- Caribbean
- ◯ Any other Black/African/Caribbean background
- ◯ Other ethnic group- Arab
- ◯ Any other ethnic group
- ◯ Prefer not to say

1

3. Smartphone Usage

* 5. Do you own a smartphone/tablet?

- Yes
- No

* 6. Do you use your smartphone/tablet to look up health information?

- Yes
- No

* 7. To your best estimation, how often do you look up health information on your smartphone?

- Several times a day
- About once a day
- 3-5 times per week
- 1-2 times per week
- Every few weeks
- Less often

* 8. Do you use any health apps? e.g. MyFitnessPal, Clue, Apple Health

- No
- Yes

1

4. General Fertility Questions

* 9. Please indicate your current menstrual status.

- ◯ Currently have regular periods
- ◯ Currently have irregular periods
- ◯ Used to have periods but don't currently
- ◯ I don't know/ It's complicated
- ◯ Other (please specify)

10. Are you using any type of birth control?

- ◯ IUD
- ◯ Contraceptive pill
- ◯ Contraceptive implant
- ◯ Barrier method e.g. male or female condom
- ◯ Contraceptive injections
- ◯ Emergency contraception
- ◯ Natural family planning i.e. fertility awareness method
- ◯ No
- ◯ Other

1

* 11. Do you use fertility/ period tracker apps?

- ◯ Clue
- ◯ Natural Cycles
- ◯ Glow
- ◯ Kindara
- ◯ Period Tracker
- ◯ Ovia
- ◯ Fertility Friend
- ◯ Cycles
- ◯ Pink Pad
- ◯ Other
- ◯ I don't use fertility/ period tracker apps

1

5. Experience of Fertility Tracking Apps

* 12. Why are you using the app(s)? (tick more than one if applicable).

- [ ] I'm trying to conceive
- [ ] As contraception
- [ ] To observe my cycle
- [ ] To inform my/ mine and my partner's fertility treatment
- [ ] Other (please specify)

13. If you use more than one fertility tracker app, please explain why.

* 14. To your best estimation, how often do you use the app(s)?

- ( ) Several times a day
- ( ) About once a day
- ( ) 3-5 days a week
- ( ) 1-2 days a week
- ( ) Every few weeks
- ( ) Less Often

* 15. Do you find the app(s) useful?

- ( ) No
- ( ) Yes

Why is it useful/ not useful

16. If you could make changes to the app(s), what would you change? e.g. add or remove any features

* 17. Do you share your data with others? (tick more than one if applicable)

- [ ] Partner
- [ ] Family and friends
- [ ] Healthcare professional
- [ ] Fertility specialist
- [ ] Online community
- [ ] I do not share my data
- [ ] Other (please specify)

1

6. I Don't Use Fertility Tracking Apps

18. If you do not use fertility tracking apps, please explain why.
Please leave this question blank if you do use fertility tracking apps.

* 19. Do you use any other method(s) to track your fertility besides an app? e.g. paper diary, website etc.

- ◯ No
- ◯ Yes

If yes, what other method(s) do you use?

1

7. Invitation for follow up interview

* 20. Thank you for completing the questionnaire! You are invited to participate in a follow up 15 minute telephone/skype interview to share more about your experience. If you are interested, I will request your consent to audio record the interview, which will be confidential and anonymous. Would you be interested in being interviewed?

- ◯ No, thank you
- ◯ Yes, my contact details are below

My email address is:

Appendix D-Social Media Posts

Fertility Network website post

An undergraduate student from the University of Aberdeen is undertaking a questionnaire and optional follow-up interview study to explore women's views of fertility tracking apps. The questionnaire should take you 12 to 15 minutes to complete and your responses will be anonymous. To take part please complete the online survey here:

https://www.surveymonkey.co.uk/r/J36YHQB

Thank you for your help!

Twitter

Could you help with some research? We would like to hear about women's views on fertility tracking apps…

https://www.surveymonkey.co.uk/r/J36YHQB

Facebook

Would you like to help with some research by completing a short survey? An undergraduate student from the University of Aberdeen is undertaking a questionnaire and optional follow-up interview study to explore women's views of fertility tracking apps. The questionnaire should take you 12 to 15 minutes to complete and your responses will be anonymous. To take part please complete the online survey here:

https://www.surveymonkey.co.uk/r/J36YHQB

Conflicting interests

The authors declare that there are no conflicting interests.

Funding

This work was conducted as a BSc (Hons) research project at the University of Aberdeen. No external funding was received.

Ethical approval

Ethical approval was sought from and granted by the College of Life Sciences and Medicine Ethics Review Board: CERB/2017/2/1426, 06/03/2017.

Guarantor

Heather May Morgan is the guarantor.

Contributorship

Katie Gambier-Ross led the design, conduct, analysis and writing. David McLernon supported substantive (fertility modelling) and methodological (statistics) work. Heather May Morgan supervised the project and supported substantive (digital health) and methodological (qualitative and mixed methods research) work. All authors contributed to this manuscript, agreed revisions based on peer review and all approved the final version.

Acknowledgement

Thanks to all participants in this research, whose contributions to the development of this field are invaluable.
